# Regulation of Med1 protein by overexpression of BAP1 in breast cancer cells

**DOI:** 10.1080/23723556.2024.2347827

**Published:** 2024-05-02

**Authors:** Hyunju Kim

**Affiliations:** Department of Health Sciences, The Graduate School of Dong-A University, Busan, South Korea

**Keywords:** Med1, BAP1, breast cancer cells, protein expression regulation, deubiquitinating enzyme

## Abstract

Med1 binds to a nuclear receptor and regulates transcription. Elevated Med1 protein expression promotes cancer growth in hormone-dependent breast and prostate cancers. Med1 protein expression was investigated by deubiquitinating enzymes (DUBs) overexpression in breast cancer cell lines. Various DNA constructs of SRT-DUBs were overexpressed in the MCF7 cell line, and Med1 protein expression was investigated by western blotting. The cell growth and in vitro invasion assay were performed in BRCA1-associated protein 1 (BAP1) wild type and mutant (C91A) overexpressed cells. Ubiquitination of the Med1 protein was observed, and Med1 protein expression and transcriptional activity were verified by various DUBs overexpressed. Although Med1 protein expression increased upon the overexpression of BAP1, it was not affected by the overexpression of BAP1 mutant (C91A). BAP1 was increased by the E2 treatment, which has an important effect on the breast cancer growth, and cell growth was decreased by BAP1 C91A overexpression. However, metastatic capacities were decreased by BAP1. In addition, the binding between the Med1 and the BAP1 protein was observed. These data suggested that BAP1 regulated Med1 protein expression in breast cancer cells and involved in cancer cell growth and metastasis by binding to Med1 protein.

## Introduction

The mediator of RNA polymerase II transcription subunit 1 mediator (Med1, also known as TRAP220) plays an important role as a coactivator of a wide range of nuclear receptors, including the estrogen receptor (ER), androgen receptor, and thyroid receptor by targeting and anchoring the complex in transcriptional regulation.^1^ Med1 regulates ER-dependent gene transcription by direct interaction with ER.^2^ Med1 protein is overexpressed in ~50% of all primary breast cancers and breast cancer cell lines.^3^ In addition, the knockdown of Med1 impairs the expression of ER-target genes and the estrogen-dependent growth of breast cancer cells,^4^ but not the expression of genes controlled by other transcriptional activators such as p53.^5^ The overexpression of Med1 in breast^5^ and prostate cancers^6^ has been described by its function as a hub for nuclear hormone receptors. In contrast, Med1 negatively modulates the expression of metastasis-related genes and is downregulated in melanoma^7^ and lung cancer.^8^ Med1 expression considerably decreases during bladder cancer progression from benign urothelium to advanced bladder cancer.^9^ Although Med1 protein expression is related to the development and metastasis of various cancers, the mechanism of regulation of Med1 protein expression is poorly understood.

Ubiquitylation plays an essential role in eukaryotic cell-signaling mechanisms by influencing protein degradation by proteasomes, altering cellular location, affecting their activity, and promoting or preventing protein interactions.^10^ Various studies have shown that activation or alterations in ubiquitination processes are associated with the pathogenesis of human carcinoma. Moreover, it has been reported that ubiquitination introduces into malignant tumors through loss of function by inactivating the mutation form, altering the protein expression, or indirectly attenuating the ubiquitination-related proteins.^11^

The protease deubiquitinating enzymes (DUBs) are known to play a role in modifying proteins by removing ubiquitin or ubiquitin-like molecules or remodeling ub-chains on target proteins and have recently been regarded as important regulators of the ubiquitination-mediated degradation and other functions.^12^ BRCA1-associated protein 1 (BAP1) is a nuclear ubiquitin hydrolase and was identified to interact with the RING finger domain of the tumor suppressor BRCA1.^13^ BAP1 belongs to a DUB with a ubiquitin carboxy-terminal hydrolase (UCH) domain and acts as a transcriptional regulator for mammalian development through cell-cycle regulation, chromatin remodeling, DNA damage response, and tumor suppression.^14^ The tumor suppressor function of BAP1 required nuclear localization and catalytic activity. Recent studies have clarified the diagnostic significance of BAP1 loss in select tumor types. Dysregulations and mutations, such as lack of deubiquitinating activity (C91A substitution) or second nuclear localization signal within the BAP1 gene, have been identified as drivers in many human cancers, such as uveal melanoma,^15^ mesothelioma,^16^ clear cell renal cell carcinoma,^17^ leukemia,^18^ and breast cancer.^19^

Therefore, in this study, Med1 protein expression was investigated by overexpression of various DUBs in the breast cancer cell line, MCF 7 cells. Cell growth was investigated using wild type (WT) and C19A mutant of BAP1, which were identified as Med1 protein regulator *in vitro.*

## Materials and methods

### Cell culture and transfection

Breast cancer cells, MCF7, lung cancer cells, A549, and HEK 293T cells were cultured in Dulbecco’s Modified Eagle’s Medium (#12800-017, Gibco), containing 10% fetal bovine serum (FBS, Gibco) and 1% penicillin-streptomycin (P/S). The cells were maintained in a 5% CO_2_ incubator at 37°C. Various full-length human DUB plasmids were cloned into the pDEST-CMV6 vector, and the plasmids are as follows: ubiquitin-specific peptidase 3 (USP3), USP21, USP22, USP25, USP28, USP28, USP35, USP36, USP39, UCH-L1, UCH-L5, and BAP1. To generate catalytic residue mutations, site-directed mutagenesis PCR (Polymerase Chain Reaction) was performed using the site-directed mutagenesis kit (#A14604, Invitrogen) according to the manufacturer’s protocol. The plasmids were as follows: USP21 C211A, USP35 C434A, and BAP1C91A. For DUBs overexpression, DUB plasmids were transfected using Lipofectamine 2000 reagent (#11668-019, Invitrogen), according to the manufacturer’s protocol.

### Western blotting and immunoprecipitation

MCF7 and HEK 293T cells were lysed in lysis buffer (50 mM Tris-HCl, pH 7.5, 1 mM EDTA, 10% glycerol, 300 mM NaCl, and 1% Triton-100), and then supernatants were collected. Protein was quantified followed by Western blotting. Membranes were blocked with TBS-T (20 mM Tris-HCl, pH 7.5, 0.05% Tween 20, and 150 mM NaCl) containing 5% skim milk or 5% bovine serum albumin for 1 h and incubated with primary antibodies overnight at 4°C. Primary antibodies used include anti-Ub (#SC-166553 m, Santa Cruz Biotechnology, Santa Cruz, CA), anti-SRT (TFIGAIATDT epitope), anti-BAP1 (#SC-28383, Santa Cruz Biotechnology, Santa Cruz, CA), anti-Med1 (#SC-8998, Santa Cruz Biotechnology, Santa Cruz, CA), and anti-β-actin (#SC-8432, Santa Cruz Biotechnology, Santa Cruz, CA). Then, the membrane was incubated with the secondary antibody for 2 h, and the blot was detected using ECL (enhanced chemiluminescence) reagent solution (#LF-QC0101, Young-In Frontier Seoul, Korea).

Immunoprecipitation (IP) experiments were performed to investigate protein interactions. Cell lysates were incubated with antibody (anti-Med1) overnight at 4°C and protein A/G PLUS-Agarose beads (# SC-2003, Santa Cruz Biotechnology, Santa Cruz, CA) for 2 h, and then Western blotting was performed.

### Luciferase reporter assay

MCF7 cells were transfected with the ERE-Luc reporter luciferase plasmid and the indicated plasmids (pDEST-CMV6-DUBs). A Renilla luciferase reporter control vector was co-transfected to normalize the luciferase activity. After transfection, cells were harvested, and a dual-luciferase reporter assay was performed. Luciferase activity was measured following the manufacturer’s protocol (Promega, Madison, WI). The relative luciferase activity was determined by dividing the firefly luciferase activity by the Renilla luciferase activity.

### Quantitative RT-PCR (qPCR)

Total RNA extraction was performed according to the manufacturer’s protocol for TRIzol reagent (#10296010, Ambion, ThermoFisher). Quantitative RT-PCR (qPCR) was performed using an SYBR Green PCR Master Mix (Takara Bio Inc., Bio Inc., Shiga) and an ABI Prism 7500 Real-Time PCR system (Applied Biosystems). β-actin transcript was used as an internal control for all samples, and gene-specific mRNA levels were normalized to β-actin mRNA levels. Expression of each mRNA was determined using the 2-^ΔΔCT^ threshold cycle method. The following primers were used: Med1: forward 5’-AGTATCATGGGCTCAGCTCC-3’ and reverse 5’-GGTGAGCCCATCATCGACAATTC-3’; BAP1: forward 5’-CCACAAGTCTCAA GAGTCACAG-3‘ and reverse 5’-CTGCACCATCTGTGTGGTT-3’; uPAR: forward 5’-CGGGCTCAATGGTTGCCA-3‘ and reverse 5’-CAGAGTGAGCGTTCGTGAGTG-3’; β-actin: forward, 5’-GCTCCCTCCTGAGCGCAAG-3‘ and reverse, 5’-CATCTGCTGGAAGGGTGGACA-3’.

### In vitro migration and invasion assays

Cell migration assays were performed using multi-chamber wells separated from complete culture medium in the bottom chamber by a polyethylene terephthalate membrane filter (pore size 8 μm) (Neuro Probe, Inc., Gaithersburg, MD). About 1 × 10^5^ cells resuspended in serum-free medium were transferred to the upper chamber, and complete culture medium was incubated in the lower chamber. After incubation for 18 h, the cells that migrated under the membrane filter were fixed with cold methanol for 20 min, and the migrated cells were stained with crystal violet. The stained cells were read for density at 595 nm using a microplate reader.

Cell invasion assays were performed using Boyden chambers with Matrigel pre-coated filter inserts (pore size 8 μm) (Costar, Corning, NY). A suspension of 1 × 10^5^ cells in medium was added to the upper chamber, and the lower chamber was filled with medium containing 10% FBS. After culturing for 18 h, invaded cells in the membrane were fixed and stained with hematoxylin and eosin (H&E). Stained cells were photographed and counted in three randomly selected fields (100× magnification).

### Cell proliferation assay

About 1 × 10^4^ cells were cultured in 6-well plates and allowed to proliferate for 6 d. The medium was changed every 2 d. After 6 d, the cells were collected, stained with trypan blue, and counted to measure proliferation. The MCF7 cells were seeded and treated each 10 ng of EGF (#PHG0311, Invitrogen), E2 (#E4389, Sigma-Aldrich), and TGF-β for 24 h and then harvested. The total cell lysates were immunoblotted with anti-Med1 and anti-BAP1.

### Statistical analysis

Densitometric analysis was performed using ImageJ. The paired *t*-test and two-way ANOVA were performed using GraphPad Prism version 5 (GraphPad Software, La Jolla, CA, USA), and *p*-values of *p < .05, **p < .01, and ***p < .001 were deemed significant. All results shown are representative data of at least three independent experiments and are presented as the mean ± standard error of the mean (SEM).

## Results

### Identification of DUBs regulating the expression of Med1 protein

**Figure 1.**
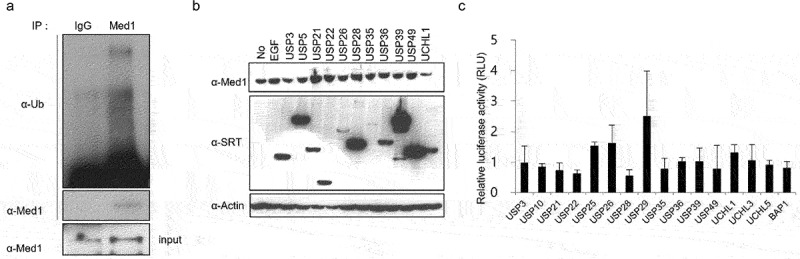
The Med1 expression was regulated through DUBs overexpression.

To determine whether the ubiquitin mechanism is involved in the regulation mechanism of Med1 protein expression, ubiquitination assay was performed in HEK 293T cells. Ubiquitin of the Med1 protein was observed in the immunoblotting with ubiquitin antibody after IP using the Med1 antibody (Figure 1a). Next, Med1 protein expression was investigated by DUBs overexpression using various DUB plasmids in MCF7 cells. It was observed that the expression of Med1 was increased by overexpression of DUBs, such as USP21, USP22, USP28, and USP49, compared with no-transfected control group. However, Med1 protein expression was decreased by UCH-L1 (Figure 1b). Abnormal expression of UCHL1 has been reported to be associated with cancer.^20,21^ These results showed that Med1 protein expression was differently regulated by overexpression of various DUBs. In order to observe the transcriptional activity of Med1, a transcriptional coregulator by overexpression of DUBs, an ERE-luciferase assay was performed. ERE-luciferase activity was not significantly affected upon overexpression of the various DUBs. Overexpression of USP29 elevated ERE-luciferase activity, whereas Med1 protein did not exhibit any affect. In addition, Med1 protein expression was decreased by UCHL1 overexpression, but ERE-transcriptional activity was not changed (Figure 1c).

To determine Med1 ubiquitination, the total 293T cell lysates were performed immunoprecipitation (IP) with anti-Med1 antibody or anti-IgG as negative control, and immunoblotting with anti-Ub antibody and anti-Med1 (A). Several DUB DNA plasmids were transfected into MCF7 breast cancer cell. The Med1 protein expression was confirmed by western blotting using anti-Med1 and anti-SRT to compare the level of control vector transfected cells and normalized with MCF7 control cells (B). The transcriptional activity of Med1 was measured by ERE-Luc reporter luciferase plasmid (C). All experiments were repeated three times, and the presented data are the mean ± standard error of the mean (SEM). * *p* < .05, ** *p* < .01, compared with MCF7 control cells.

### Regulation of Med1 protein expression and function by BAP1 overexpression

**Figure 2.**
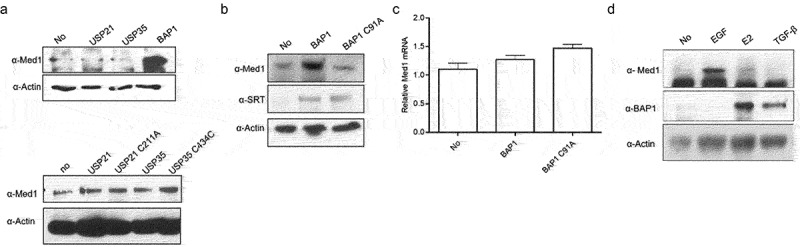
Med1 protein and mRNA expression through BAP1 overexpression.

Overexpressions of USP21, USP35, and BAP1 differently regulate Med1 protein expression and transcriptional capacity in MCF7 cells. Med1 protein expression was highly increased by BAP1 overexpression, whereas it did not affect USP21 and USP35 (Figure 2a). To confirm these results, a mutant DNA plasmid with inhibition of the catalytic activity was used. The expression of Med1 protein was not affected by USP21 C211A and USP35 C434A mutant overexpression (Figure 2a). These data suggest that USP21 and USP35 do not regulate Med1 protein expression through catalytic activity. Med1 protein expression was increased by BAP1 wild-type overexpression in MCF7 cells, whereas it was decreased by BAP1 C91A mutant overexpression (Figure 2b). A qPCR result revealed that Med1 mRNA expression was not affected by the overexpression of the BAP1WT or BAP1 C91A mutant (Figure 2c). These results demonstrated that Med1 protein regulation by BAP1 was regulated at the protein level and not at transcriptional level. Next, to verify the relationship between Med1 and BAP1 protein expression, it was treated with EGF, E2, and TGF-β, which are known to play an important role in breast cancer growth regulation. Med1 protein and mRNA (data not shown) were increased by EGF treatment, but they were not increased by E2 and TGF-β. On the other hand, BAP1 was significantly increased by E2 and TGF treatment (Figure 2d). These results addressed that the regulation of Med1 protein expression by BAP1 is regulated at the protein level and is regulated by different mechanisms from growth factors.

To determine Med1 protein expression, MCF7 cells were transfected with SRT-USP21, SRT-USP35, SRT-BAP1 wild-type (WT) or SRT-BAP1 C91A mutant-type (MT) DNA plasmids. The total cell lysates were immunoblotted with the anti-Med1 and anti-SRT. Expression of β-actin was used as a loading control. (A, B) Med1 mRNA expression was performed by real-time PCR. The Med1 mRNA expression was normalized with control vector-introduced cells (C). The MCF7 cells were harvested for 24 h after treatment with 10 ng EGF, E2, TGF-β, and then the total cell lysates were immunoblotted with anti-Med1 and anti-BAP1 (D). The values shown are the means of three independent experiments. The presented data are the means ± standard error of mean (SEM). *p < .05, **P < .01 compared with MCF7 control cells.

### Med1 is associated with BAP1

**Figure 3.**
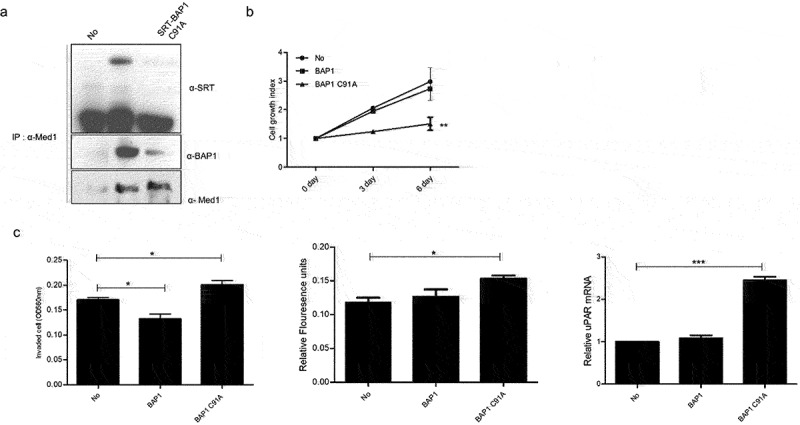
Med1 interacted with BAP1 and regulated growth and metastatic ability.

To investigate the interaction between Med1 and BAP1 protein, co-immunoprecipitation was performed in the BAP1 overexpressing cell line, BAP1 WT and BAP1 C91A mutant were overexpressed, and IP was performed using an anti-Med1 antibody in BAP1 or BAP1 C91A overexpressed cells. Immunoblotting was performed using anti-SRT and anti-BAP1 antibodies. Med1 was observed to bind with BAP1. However, the BAP1 C91A mutant was observed to bind weakly compared with the BAP1 control (Figure 3a). These results demonstrate that BAP1 is associated with Med1, but not with the BAP1 catalytic mutant.

To determine Med1 and BAP1 interaction, 293T cells were transfected with BAP1 or BAP1 C91A DNA plasmids for 36 h and then the cell lysates were immunoprecipitated with anti-Med1 antibody and subsequently immunoblotted with anti-SRT antibody or immunoblotted with anti-BAP1 (A). Cell proliferation was measured by counting the number of trypan-blue staining cells at 3 d and 6 d after transfecting SRT-BAP1 or SRT-BAP1 C91A DNA plasmids into MCF7 cells. All proliferation assays were repeated three times (B). The Matrigel cell invasion assay was performed using SRT-BAP1 WT or SRT-BAP1 C91A DNA plasmids and compared with MCF7 control cells. After incubating the cell with Matrigel-coated filters for 24 h, the cells on the undersurface of the filter were staining with hematoxylin and eosin (H&E stain). The invaded cell measured OD values using spectrophotometer. The cell was starved in serum-free media for 24 h before being to the manufacturer’s instructions. The migrated cells were stained, and OD values were measured using spectrophotometer. Quantitative RT-PCR (qPCR) analysis was performed using gene-specific primer in BAP1 or BAP1 C91A DNA plasmids transfected cells to confirm the observed alteration of uPAR target gene expression. Later, the mRNA level of each gene was normalized to the level of β-actin mRNA. The relative mRNA was compared with MCF7 control cells (C).

### BAP1 overexpression regulates cell growth and cancer metastasis in vitro

Med1 regulates the transcription of various genes and plays a role in cell growth and cancer metastasis. Therefore, to investigate whether the overexpression of BAP1 affects proliferation, metastasis by Med1 protein expression, cell proliferation, and invasion assays were performed in MCF7 breast cancer cells. There was no difference in cell growth in the BAP1 overexpressing cells compared with the control in the MCF7 cell. However, cell growth was reduced in the BAP1 C91A mutant overexpressing cells (Figure 3b). In a previous report, BAP1 increased cell growth, whereas abnormal activity of BAP1 inhibited cell growth in several cancers. It was observed that overexpression of BAP1 WT slightly decreased invasion ability but increased cell migration. The overexpression of BAP1 C91A mutants was observed to increase metastatic ability, and mRNA level of the urokinase-type plasminogen activator receptor (uPAR) gene, a metastasis-related gene, was significantly increased by overexpression of the BAP1 mutant (Figure 3d). Many studies have reported that inhibition of Med1 expression increased cancer metastasis and is associated with uPAR gene expression. Therefore, these results demonstrate that cell growth is regulated by overexpression of BAP1, and metastatic potential can be modulated by aberrant activation of BAP1 or Med1 protein expression.

### Regulation of Med1 protein expression in lung cancer cells

**Figure 4.**
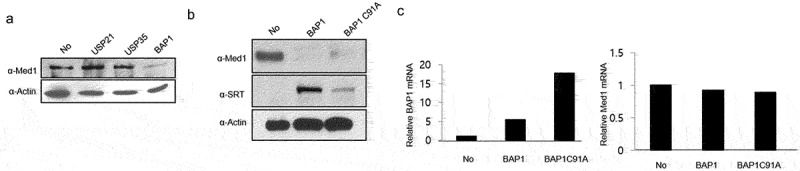
Med1 protein and mRNA expression through BAP1 overexpression in lung cancer cells.

To investigate the expression of Med1 by overexpressing BAP1 in non-small-cell lung cancer, A549 cells, USP21, USP35, and BAP1 were overexpressed. Unlike MCF7, Med1 protein expression was decreased by BAP1 overexpression (Figure 4a). Additionally, overexpressing BAP1 C91A mutant led to a reduced inhibition of Med1 protein expression (Figure 4b). It was verified through qPCR that this did not affect Med1 mRNA expression of Med1 (Figure 4c). These findings suggest that the regulation of Med1 by BAP1 primarily occurs at the protein level and not at the transcription level. The relationship between BAP1 and Med1 expression may be context-dependent and could differ in other cancer cell lines or types.

To determine Med1 protein, A549 cells were transfected with SRT-USP21, SRT-USP35, SRT-BAP1 wild type (WT) or SRT-BAP1 C91A mutant type (MT) DNA plasmids. The total cell lysates were immunoblotted with the anti-Med1 and anti-SRT. Expression of β-actin was used as a loading control (A, B). Med1 and BAP1 mRNA expressions were performed by real-time PCR. Expression of β-actin was used as a loading control. The Med1 and BAP1 mRNA expressions were normalized with control vector introduced cells (C).

## Discussion

Med1 (also known as Trap220 or DRIP205) is a subunit of the mediator complex. Recent studies have suggested that Med1 protein expression plays a role in tumorigenesis and cancer metastasis. The deficiency of the Med1 protein functions as a tumor suppressor gene and inhibits invasion and metastasis in lung carcinoma^8^ and melanoma cells.^22^ Loss of Med1 significantly decreases the proliferation of prostate cancer cells and breast cancer.^23^ In contrast, a deficiency of Med1 protects hepatocytes from chemical carcinogen-induced hepatocarcinogenesis^24^ and osteoblastoma.^25,26^ Some kinases such as AKT, ERK, and DNA-PK mediate the phosphorylation of Med1 and enhance Med1 association with transcriptional regulation.^6,27,27^ Overexpression of Med1 abrogated the effects of miR-1 on cell proliferation.^28^ However, there have been few studies on the mechanism of Med1 protein expression regulation. In this study, it was investigated that overexpression of various DUBs regulates the expression of the Med1 protein. Some DUBs such as USP21 and USP35 WT increased the Med1 protein expression, but they did not regulate the Med1 protein expression by overexpression of catalytic mutant forms (USP21 C211A, and USP35 C434A). These results explain the UPS21 and UPS35 affect Med1 protein expression by indirect regulation mechanism and the underlying mechanism is unclear. However, Med1 protein was increased by the overexpression of BAP1 and decreased by the overexpression of BAP1 C91A. Various studies have reported that BAP1 acts as a tumor suppressor by binding BRCA1 in breast cancer^13^ and BAP1 cancer syndrome, in which metastatic breast cancer develops with loss of BAP1 protein expression.^29^ This results suggest that the overexpression of BAP1 in breast cancer is involved in the transcriptional regulation of ER by stabilizing the Med1 protein. It also affected the growth and metastasis of cancer cells. uPAR expression is elevated during tissue remodeling and in many human cancers.^30^ It has been reported that inhibition of Med1 expression in lung cancer cell lines increases uPAR transcriptional activity.^8^ Inhibition of Med1 by overexpression of BAP1 C91A affects uPAR transcription. BAP1 and Med1 are located in the nucleus, and they modulated transcriptional activity. In addition, an examination of the interaction between Med1 and BAP1 proteins showed that Med1 and BAP1 proteins are binding and are involved in the development and metastasis of cancer (Figure 3). Although the binding of Med1 and BAP1 involved in the cancer growth or metastasis, mechanisms are still unclear. However, in the future, the results of this study will contribute to various research fields, such as the mechanism of regulating the expression of target genes by the binding of Med1 and BAP1 and the growth and metastasis of cancer by Med1 and BAP1. In addition, it may contribute to various studies, such as determining the role of Med1 protein expression through post-translational phosphorylation of Med1, which forms or maintains an active chromatin structure, in binding to BAP1 and ubiquitination. Further studies can investigate whether Med1 phosphorylation affects the binding ability of BAP1 protein and the role in cancer cell growth and metastasis capacity.

Breast cancer is the most common cancer in women worldwide, and the risk increases with age, reproductive factors, personal or family history of breast disease, genetic predisposition, and environmental factors.^31^ Two key target molecules have been identified in breast cancer pathogenesis namely ERα, which is expressed in approximately 70% of invasive breast cancers, and epidermal growth factor 2 (formerly HER2 or HER2/neu), which is overexpressed in approximately 20% of breast cancers. The Med1 gene is located within the 17q12 region of chromosome 17, also known as the HER2 amplicon, which is amplified in approximately 20%–25% of breast tumors and binds directly to the ER through the LXXLL motif.^32^ This suggests that Med1 is a key regulator of cellular processes involved in breast tumor growth, metastasis, cancer stem cell formation, and treatment resistance. Thus, Med1 and BAP1 can serve as biomarkers for cancer development or metastasis, and targeting agents serve as potential gene therapeutic targets in various cancers.

## Data Availability

The data used to support the findings of this study are included within the article.

## References

[1] Ito M, Roeder RG. The TRAP/SMCC/Mediator complex and thyroid hormone receptor function. Trend Endocrinol Metabol. 2001;12(3):127–7. doi:10.1016/S1043-2760(00)00355-6. 11306338

[2] Zhang X, Krutchinsky A, Fukuda A, Chen W, Yamamura S, Chait BT, Roeder RG. MED1/TRAP220 exists predominantly in a TRAP/Mediator subpopulation enriched in RNA polymerase II and is required for ER-mediated transcription. Mol Cell. 2005;19(1):89–100. doi:10.1016/j.molcel.2005.05.015. 15989967

[3] Zhu Y, Qi C, Jain S, Le Beau MM, Espinosa R, Atkins GB, Lazar MA, Yeldandi AV, Rao MS, Reddy JK. Amplification and overexpression of peroxisome proliferator activated receptor binding protein (PBP/PPARBP) gene in breast cancer. Proc Natl Acad Sci USA. 1999;96(19):10848–10853. doi:10.1073/pnas.96.19.10848. 10485914 PMC17971

[4] Leonard M, Zhang X. Estrogen receptor coactivator mediator subunit 1 (MED1) as a tissue-specific therapeutic target in breast cancer. J Zhejiang Univ-sc B. 2019;20:381–390. 10.1631/jzus.B1900163 PMC6568227 31090264

[5] Frade R, Balbo M, Barel M. RB18A regulates p53-dependent apoptosis. Oncogene. 2002;21(6):861–866. doi:10.1038/sj.onc.1205177. 11840331

[6] Jin F, Irshad S, Yu W, Belakavadi M, Chekmareva M, Ittmann MM, Abate-Shen C, Fondell JD. ERK and AKT signaling drive MED1 overexpression in prostate cancer in association with elevated proliferation and tumorigenicity MED1 overexpression in prostate cancer. Mol Cancer Res. 2013;11(7):736–747. doi:10.1158/1541-7786.MCR-12-0618. 23538858 PMC5838364

[7] Ndong J, Jean D, Rousselet N, Frade R. Down‐regulation of the expression of RB18A/MED1, a cofactor of transcription, triggers strong tumorigenic phenotype of human melanoma cells. Int J Cancer. 2009;124(11):2597–2606. doi:10.1002/ijc.24253. 19243021

[8] Kim H-J, Roh MS, Son CH, Kim AJ, Jee HJ, Song N, Kim M, Seo S-Y, Yoo YH, Yun J. et al. Loss of Med1/TRAP220 promotes the invasion and metastasis of human non-small-cell lung cancer cells by modulating the expression of metastasis-related genes. Cancer Lett. 2012;321(2):195–202. doi:10.1016/j.canlet.2012.02.009. 22342682

[9] Klümper N, Syring I, Vogel W, Schmidt D, Müller SC, Ellinger J, Adler D, Brägelmann J, Perner S. Mediator complex subunit MED1 protein expression is decreased during bladder cancer progression. Front Med. 2017;4:30. doi:10.3389/fmed.2017.00030. PMC5355444 28367434

[10] Swatek KN, Komander D. Ubiquitin modifications. Cell Res. 2016;26(4):399–422. doi:10.1038/cr.2016.39. 27012465 PMC4822133

[11] Sun T, Liu Z, Yang Q. The role of ubiquitination and deubiquitination in cancer metabolism. Mol Cancer. 2020;19(1):1–19. doi:10.1186/s12943-020-01262-x. 33004065 PMC7529510

[12] He M, Zhou Z, Shah AA, Zou H, Tao J, Chen Q, Wan Y. The emerging role of deubiquitinating enzymes in genomic integrity, diseases, and therapeutics. Cell & Biosci. 2016;6(1):1–15. doi:10.1186/s13578-016-0127-1. PMC5168870 28031783

[13] Jensen DE, Proctor M, Marquis ST, Gardner HP, Ha SI, Chodosh LA, Ishov AM, Tommerup N, Vissing H, Sekido Y. et al. BAP1: a novel ubiquitin hydrolase which binds to the BRCA1 RING finger and enhances BRCA1-mediated cell growth suppression. Oncogene. 1998;16(9):1097–1112. doi:10.1038/sj.onc.1201861. 9528852

[14] Mashtalir N, Daou S, Barbour H, Sen NN, Gagnon J, Hammond-Martel I, Dar H, Therrien M, Affar E. Autodeubiquitination protects the tumor suppressor BAP1 from cytoplasmic sequestration mediated by the atypical ubiquitin ligase UBE2O. Mol Cell. 2014;54(3):392–406. doi:10.1016/j.molcel.2014.03.002. 24703950

[15] Harbour JW, Onken MD, Roberson ED, Duan S, Cao L, Worley LA, Council ML, Matatall KA, Helms C, Bowcock AM. et al. Frequent mutation of BAP1 in metastasizing uveal melanomas. Science. 2010;330(6009):1410–1413. doi:10.1126/science.1194472. 21051595 PMC3087380

[16] Testa JR, Cheung M, Pei J, Below JE, Tan Y, Sementino E, Cox NJ, Dogan AU, Pass HI, Trusa S. et al. Germline BAP1 mutations predispose to malignant mesothelioma. Nat Genet. 2011;43(10):1022–1025. doi:10.1038/ng.912. 21874000 PMC3184199

[17] Peña-Llopis S, Vega-Rubín-de-Celis S, Liao A, Leng N, Pavía-Jiménez A, Wang S, Yamasaki T, Zhrebker L, Sivanand S, Spence P. et al. BAP1 loss defines a new class of renal cell carcinoma. Nat Genet. 2012;44(7):751–759. doi:10.1038/ng.2323. 22683710 PMC3788680

[18] Wang L, Birch NW, Zhao Z, Nestler CM, Kazmer A, Shilati A, Blake A, Ozark PA, Rendleman EJ, Zha D. et al. Epigenetic targeted therapy of stabilized BAP1 in ASXL1 gain-of-function mutated leukemia. Nat Cancer. 2021;2(5):515–526. doi:10.1038/s43018-021-00199-4. 35122023

[19] Qin J, Zhou Z, Chen W, Wang C, Zhang H, Ge G, Shao M, You D, Fan Z, Xia H. et al. BAP1 promotes breast cancer cell proliferation and metastasis by deubiquitinating KLF5. Nat Commun. 2015;6(1):1–12. doi:10.1038/ncomms9471. PMC4598844 26419610

[20] Liu Z, Meray RK, Grammatopoulos TN, Fredenburg RA, Cookson MR, Liu Y, Logan T, Lansbury PT. Membrane-associated farnesylated UCH-L1 promotes α-synuclein neurotoxicity and is a therapeutic target for Parkinson’s disease. Proc Natl Acad Sci USA. 2009;106(12):4635–4640. doi:10.1073/pnas.0806474106. 19261853 PMC2651203

[21] Liu Y, Lashuel HA, Choi S, Xing X, Case A, Ni J, Yeh L-A, Cuny GD, Stein RL, Lansbury PT. et al. Discovery of inhibitors that elucidate the role of UCH-L1 activity in the H1299 lung cancer cell line. Chem Biol. 2003;10(9):837–846. doi:10.1016/j.chembiol.2003.08.010. 14522054

[22] Gade P, Singh AK, Roy SK, Reddy SP, Kalvakolanu DV. Down‐regulation of the transcriptional mediator subunit Med1 contributes to the loss of expression of metastasis‐associated dapk1 in human cancers and cancer cells. Int J Cancer. 2009;125(7):1566–1574. doi:10.1002/ijc.24493. 19521987 PMC4010141

[23] Chen Z, Zhang C, Wu D, Chen H, Rorick A, Zhang X, Wang Q. Phospho‐MED1‐enhanced UBE2C locus looping drives castration‐resistant prostate cancer growth. Embo J. 2011;30(12):2405–2419. doi:10.1038/emboj.2011.154. 21556051 PMC3116285

[24] Rasool R RU, Natesan R, Deng Q, Aras S, Lal P, Effron SS, Mitchell-Velasquez E, Posimo JM, Carskadon S, Baca SC. et al. CDK7 inhibition suppresses castration-resistant prostate cancer through MED1 inactivation. Cancer Discov. 2019;9(11):1538–1555. doi:10.1158/2159-8290.CD-19-0189. 31466944 PMC7202356

[25] Belakavadi M, Pandey PK, Vijayvargia R, Fondell JD. MED1 phosphorylation promotes its association with mediator: implications for nuclear receptor signaling. Mol Cell biol. 2008;28(12):3932–3942. doi:10.1128/MCB.02191-07. 18391015 PMC2423130

[26] Jia Y, Viswakarma N, Reddy JK. Med1 subunit of the mediator complex in nuclear receptor-regulated energy metabolism, liver regeneration, and hepatocarcinogenesis. Gene Expr. 2014;16(2):63. doi:10.3727/105221614X13919976902219. 24801167 PMC4093800

[27] Schiano C, Rienzo M, Casamassimi A, Napoli C. Gene expression profile of the whole mediator complex in human osteosarcoma and normal osteoblasts. Med Oncol. 2013;30(4):1–6. doi:10.1007/s12032-013-0739-9. 24101134

[28] Jiang C, Chen H, Shao L, Wang Q. MicroRNA-1 functions as a potential tumor suppressor in osteosarcoma by targeting Med1 and Med31. Oncol Rep. 2014;32(3):1249–1256. doi:10.3892/or.2014.3274. 24969180

[29] Blatnik A, Ribnikar D, Šetrajčič Dragoš V, Novaković S, Stegel V, Grčar Kuzmanov B, Boc N, Perić B, Škerl P, Klančar G. et al. BAP1-deficient breast cancer in a patient with BAP1 cancer syndrome. Emerg Cancer Ther. 2022;29(5):921–927. doi:10.1007/s12282-022-01354-0. PMC9385750 35381901

[30] Shah R. Pathogenesis, prevention, diagnosis, and treatment of breast cancer. World J Clin Oncol. 2014 Aug 8;5(3):283. doi:10.5306/wjco.v5.i3.283. 25114845 PMC4127601

[31] Smith HW, Marshall CJ. Regulation of cell signalling by uPAR. Nat Rev Mol Cell Biol. 2010 Jan;11(1):23–36. doi:10.1038/nrm2821. 20027185

[32] Waks AG, Winer EP. Breast cancer treatment: a review. JAMA. 2019 Jan 22;321(3):288–300. doi:10.1001/jama.2018.19323. 30667505

